# Supplementary material for: Results of a 2021 French National Survey on Management of Patients with Advanced Stage Epithelial Ovarian Cancer
Source: J Clin Med. 2021 Oct 21;10(21):4829. doi: 10.3390/jcm10214829 (PMC8585030; doi:10.3390/jcm10214829)
Supplement: Supplementary file 1 [file jcm-10-04829-s001.zip › jcm-1406051-supplementary.pdf]

Supplementary Figure S1: Final Survey

Socio-demographic and centre data of the participant

Age
Gender
Length of practice
Specialty
Geographic location of practice
Number of ovarian cancers managed per year by your institution
Number of surgeons operating on ovarian cancers in your facility
Number of trials opened for ovarian cancer (including early stage)
Possession of ESGO accreditation
Extension work-up performed
Review of imaging and pathology data
Use of O-RADS MRI score in your centre
Data on files presented in the multidisciplinary team
Members of the multidisciplinary team
Presence of a geriatric oncologist

Pathological/genetic data

Condition of anatomo-pathological review
Time (months) to obtain an oncogenetic consultation
Conditions relating to searching for and obtaining the somatic and/or germline BRCA mutation and/or HRD

Surgery

Mean time from first consultation to cytoreduction surgery for patients who are operable at the outset
Percentage of patients who have first surgery
Use of standardised ESGO-type operative report?
Use of resectability scores for peritoneal carcinosis and method of evaluation
Criteria for deciding on primary surgery
Data on laparoscopy performance
Data on the realisation of the lymph node surgery: imaging, use of LIONS criteria
Types of surgical procedures performed by the practitioner
Data on CHIP and PIPAC

Chemotherapy

Average time between first consultation and first chemotherapy
Types of treatment performed according to BRCA status, surgery (neo-adjuvant, adjuvant, CCO or CC1): chemotherapy, bevacizumab, PARP inhibitors

Follow-up

Monitoring tools used

Supplementary Table S1. Percentage of response and specialty of participants for each section

Parameters	Respondents (%)
Answers to pathological questions – no [1] (%)	34 (37.8)
Medical oncologist - no. (%)	8 (23.5)
Obstetrician-gynaecologist - no. (%)	11 (32.4)
Surgical oncologist - no. (%)	10 (29.4)

Geriatric oncologist - no. (%)	0 (0)
Pathologist - no. (%)	5 (14.7)
Answers to surgery questions - no (%)	83 (77.6)
Medical oncologist - no. (%)	5 (6.0)
Obstetrician-gynaecologist - no. (%)	40 (48.2)
Surgical oncologist - no. (%)	37 (44.6)
Geriatric oncologist - no. (%)	0 (0)
Pathologist - no. (%)	1 (1.2)
Answers to medical oncology questions - no (%)	33 (30.8)
Medical oncologist - no. (%)	19 (57.6)
Obstetrician-gynaecologist - no. (%)	6 (18.2)
Surgical oncologist - no. (%)	7 (21.2)
Geriatric oncologist - no. (%)	1 (3.0)
Pathologist - no. (%)	0 (0)

[1] Number of respondents.

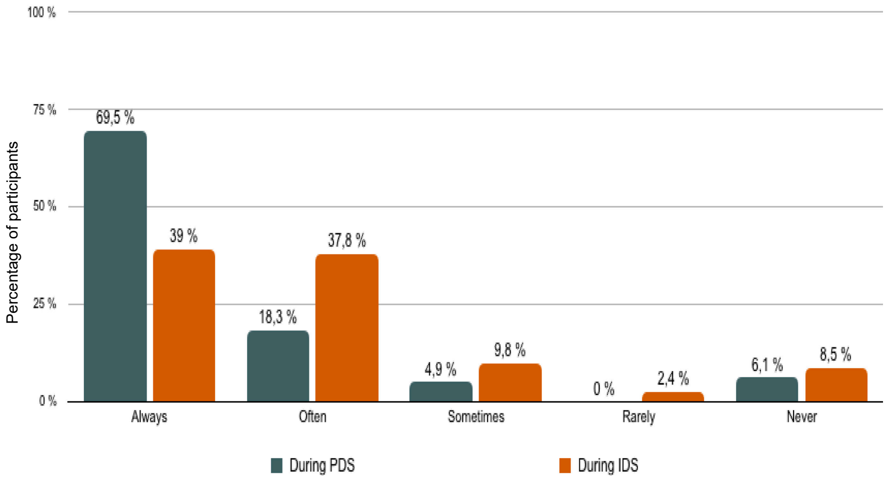

Supplementary Figure S2. Percentage of participants according to their application of the LION criteria during PDS and/or IDS.
